# Antinociceptive effect of methanol extract of *Dalbergia sissoo* leaves in mice

**DOI:** 10.1186/s12906-017-1565-y

**Published:** 2017-01-23

**Authors:** Md. Abdul Mannan, Ambia Khatun, Md. Farhad Hossen Khan

**Affiliations:** Department of Pharmacy, Stamford University Bangladesh, 51, Siddeswari Road, Dhaka, 1217 Bangladesh

**Keywords:** *Dalbergia sissoo*, Antinociceptive, Opioid receptors

## Abstract

**Background:**

*Dalbergia sissoo* DC. (Family: Fabaceae) is a medium to large deciduous tree, is locally called “shishu” in Bangladesh. It is used to treat sore throats, dysentery, syphilis, bronchitis, inflammations, infections, hernia, skin diseases, and gonorrhea. This study evaluated the antinociceptive effect of the methanol extract of *D. sissoo* leaves (MEDS) in mice.

**Methods:**

The extract was assessed for antinociceptive activity using chemical and heat induced pain models such as hot plate, tail immersion, acetic acid-induced writhing, formalin, glutamate, and cinnamaldehyde test models in mice at the doses of 100, 200, and 400 mg/kg (p.o.) respectively. Morphine sulphate (5 mg/kg, i.p.) and diclofenac sodium (10 mg/kg, i.p.) were used as reference analgesic drugs. To confirm the possible involvement of opioid receptor in the central antinociceptive effect of MEDS, naloxone was used to antagonize the effect.

**Results:**

MEDS demonstrated potent and dose-dependent antinociceptive activity in all the chemical and heat induced mice models (*p* < 0.001). The findings of this study indicate that the involvement of both peripheral and central antinociceptive mechanisms. The use of naloxone verified the association of opioid receptors in the central antinociceptive effect.

**Conclusions:**

This study indicated the peripheral and central antinociceptive activity of the leaves of *D. sissoo*. These results support the traditional use of this plant in different painful conditions.

## Background

*Dalbergia sissoo* DC. (Family: Fabaceae) is a medium to large deciduous tree, is commonly known as sisu, sheesham, tahli, tali, jag at different parts of the world and is locally called “shishu” in Bangladesh. It is usually used to treat sore throats, dysentery, syphilis, and gonorrhoea [1]. It is used in conditions like blood purifier, leprosy, headaches, bronchitis, inflammations, infections, hernia, and skin diseases [2]. It has been also reported to use as stimulant, astringent [3]; colorectal cancer and bacterial infections [4]. Juice of the leaves is useful for anthelmintic, eye and nose diseases. It also used in scabies, burning sensation, scalding urine and digestive disorders [5–8]. It is used as expectorant, anti-emetic, leucoderma, ulcers, and gout like medicinal properties [9]. Boiled leaf filtrate is used to wash hair for removing dandruff and for long hair [10].

Isoflavones, biochanin-A, muningin, sissotrin, amyrin, stigmasterol have been isolated from the aerial parts of *Dalbergia sissoo* Roxb [11]. The stem-bark has been reported to contain dalberjenone, dalbergin and methyl dalbergin and a new 4-phenyl chromene, dalbergichromene [12]. The mature pods contain a new isoflavone glucoside identified as caviunin 7-0-gentiobioside [13], isoflavone diglucoside recognize as isocaviunin 7-0-gentiobioside [14]. The root bark of *Dalbergia sissoo* contain a chalcone, 2, 3- dimethoxy-40-g, g-dimethylallyloxy-20-hydroxychalcone; an isoflavone, 7-g, g-dimethylallyloxy- 5-hydroxy-40-methoxyisoflavone; a flavone, 7-hydroxy-6-methoxyflavon; a isoflavone, biochanin A and rotenoid, dehydroamorphigenin [15]. Rhamnose, galactose, and glucuronic acid have been isolated from the leave [16]. Compounds obtained from *Dalbergia sissoo* like an isoflavone, biochanin is a potent chemotherapeutic cancer preventive agent with a distinct estrogenic activity has been isolated from the fresh flowers of *Dalbergia sissoo*. Two rare glycosides such as kaempferol and quercetin rutinosides were isolated in a low yield from the flowers of *Dalbergia sissoo* [17, 18].

Pharmacological studies have evaluated the analgesic and anti-pyretic [19], anti-diabetic [20], anti-inflammatory [21] and anti-diarrheal effect of the extract [22]. The plant bark has been reported the anti-nociceptive [23], anti-oxidant [24] and anti-spermatogenic activity [25]. The oil extracted from wood scrapings of *Dalbergia sissoo* has shown dose dependent larvicidal activity against mosquitoes [26]. Anti-osteogenic activity has also been performed this crude extract of *Dalbergia sissoo* [27]. The plant extract has been used as an alternative to synthetic pesticides for termite control in buildings [28]. The seeds extract has modest analgesic and notable anti-pyretic activities [29]. The ethanolic extract of the bark of *Dalbergia sissoo* Roxb. was investigated for its activity against helminthic infections [30]. In the search for the molluscicidal activity, the plant extracts were evaluated against egg masses and adults of *Biomphalaria pfeifferi* and the snail intermediate host of *Schistosoma manson* in Nigeria [31]. In-vivo, intrastriatal injection of mitochondrial toxin in rats produce excitotoxic lesions, while chronic administration of 3-NP produces excitotoxic-like lesions regionally restricted to striatum in both rats and non-human primates [32]. It prevents central nervous system damage [33].

According to the previous study, leaves and barks of *D. sissoo* presented the antinociceptive properties [19, 23]. Based on the studies, we designed and conducted the present work to assess the effect of methanol extract of *D. sissoo* leaves with six pain models in mice and two mechanism of action study for possible activity. The present research also warrants further investigation into the complex mechanism of action of methanol extract of *D. sissoo* leaves on induced antinociception.

## Methods

### Plant material and extraction

The fresh leaves of *D. sissoo* were collected from Khanpur, Bogra, Bangladesh in the month of June 2015. The plant samples were then identified and authenticated by Sarder Nasir Uddin, Senior Scientific Officer, Bangladesh National Herbarium, Dhaka, Bangladesh. A voucher specimen number (DACB: 42317) has already been deposited in the Herbarium for further reference. The fresh leaves were dark dried for one week and grinded to a fine powder. Powdered dried leaves (300 g) were macerated with 1000 mL of methanol with occasional stirring at 25 ± 2 °C for 3 days in a beaker. The extract was then filtered using a Whatman No. 1 filter paper and a sterilized cotton filter. The solvent was completely removed by rotary evaporator (BC-R 201 Shanghai Biochemical Equipment Co. Ltd.) and 17.59 g extract (Yield 5.86%) was obtained. This extract was used for the acute toxicity and antinociceptive activity studies.

### Animals

Swiss albino mice (20–25 g) were collected from the Animal Research Branch of the International Center for Diarrheal Disease and Research, Bangladesh (ICDDR, B). Animals were kept under standard laboratory conditions (room temperature: 25.0 ± 2.0 °C, relative humidity: 55–65% and 12 h light/dark cycle) with food and water during the adaptation period. The animals were acclimated to the laboratory environment for a period of two weeks prior to performing the experiments. Mice were fasted overnight prior to the experiments. All the experimental animals were treated following the Ethical Principles and Guidelines for Scientific Experiments on Animals (1995) formulated by The Swiss Academy of Medical Sciences and the Swiss Academy of Sciences. The Ethics Committee of Stamford University Bangladesh (SUB/IAEC/16.01) approved all experimental protocols.

### Drugs and treatments

The control group orally received deionized water at the volume of 0.1 mL/mouse 30 min before the experiments. The positive control group intraperitoneally (i.p.) received standard drug morphine in hot plate, and tail immersion test at the dose of 5 mg/kg and diclofenac sodium in acetic acid-induced writhing, formalin-induced licking, glutamate-induced paw licking, and cinnamaldehyde-induced licking test at the dose of 10 mg/kg 15 min before the experiments. MEDS was administered orally at the doses of 100, 200, and 400 mg/kg (b.w.) 30 min before the experiments. To evaluate the involvement of opioid-mediated antinociceptive activity, naloxone was administered (i.p.) at the dose of 2 mg/kg 15 min before morphine sulfate (5 mg/kg, i.p.) or MEDS (100, 200, and 400 mg/kg, p.o.) administration in the hot plate and tail immersion test. Methylene blue (20 mg/kg) and glibenclamide (10 mg/kg) were intraperitoneally employed 15 min before control (Equivalent volume of deionized water, 0.1 mL/mouse, p.o.), or MEDS (100, 200, and 400 mg/kg, p.o.) administration to evaluate the involvement of cyclic guanosine monophosphate (cGMP) and ATP-sensitive K^+^ channel pathway respectively. All the doses of drugs and MEDS were prepared using deionized water.

### Acute toxicity test

Acute toxicity studies were carried on mice according to the method proposed by Ghosh, MEDS at the doses of 50, 100, 300, 1000, and 3000 mg/kg body weight were orally administered to separate groups of the mice (*n* = 5) after overnight fasting. Subsequent to administration of MEDS, the mice observed closely for the first 3 h for toxic manifestations like enlarged motor activity, salvation, convulsions, coma, and death. The observations were made at regular intervals for 24 h. The animals were observed for one week [34].

### Phytochemical screening

The crude methanolic extract of *D. sissoo* (MEDS) was qualitatively tested for the detection of alkaloids, flavonoids, saponins, tannins, glycosides, carbohydrates, reducing sugars, proteins, glucosides, terpenoids, and steroids following standard procedures [35].

### Antinociceptive activity test

#### Hot plate test

The hot plate test method was employed for the purpose of preferential assessment of possible centrally mediated analgesic effects [36]. Mice were divided into five groups of five mice each. Mice were treated with control (Equivalent volume of deionized water, 0.1 mL/mouse, p.o.), morphine as a standard drug (5 mg/kg, i.p.) or MEDS (100, 200 and 400 mg/kg, p.o.) and was placed on Eddy’s hot plate kept at a temperature of 52 ± 1 °C. A cut off period of the 20s was maintained to avoid paw tissue damage. The response in the form of forepaw licking, withdrawal symptom of the paws or jumping was recorded at 0, 30, 60, 90, and 120 min following treatment. Then, the percentage of the highest possible effect (% MPE) was calculated using the following formula:

$$ \%\ \mathrm{M}\mathrm{P}\mathrm{E} = \left[\left(\mathrm{P}\mathrm{ostdrug}\ \mathrm{latency}\right)\ \hbox{-}\ \left(\mathrm{P}\mathrm{r}\mathrm{e}\mathrm{drug}\ \mathrm{latency}\right)/\left(\mathrm{Cut}\ \mathrm{off}\ \mathrm{time}\right)\ \hbox{-}\ \left(\mathrm{P}\mathrm{r}\mathrm{e}\hbox{-} \mathrm{drug}\ \mathrm{latency}\right)\right] \times 100. $$

#### Tail immersion test

The tail immersion test is based upon the observation that morphine-like drugs selectively prolongs the reaction time of the typical tail withdrawal reflex in mice. This method was used to evaluate the central mechanism of analgesic activity. Here, the painful reactions in animals were produced by the thermal incentive that is dipping by the tip of the tail in hot water [37]. Mice were divided into five groups consisting of five mice in each group. According to the procedure, 1 to 2 cm of the tail of mice pretreated with morphine (5 mg/kg, i.p.) or MEDS (100, 200 and 400 mg/kg, p.o.) were immersed in warm water kept constant at 54 ± 1 °C. The latency between tail submersion and deflection of the tail was recorded. A latency period of the 20s was maintained to avoid tail tissue damage in mice. The latency period of the tail-withdrawal response was taken as the indicator of antinociception and was determined at 0, 30, 60, 90, and 120 min after the administration of the morphine and MEDS. Then, the % MPE was calculated from using the same formula used in hot plate test.

#### Acetic acid-induced writhing test

This test was performed to estimate the peripheral antinociceptive activity of MEDS in chemical-induced pain. The animals were divided into five groups (*n* = 5). The animals were treated with control (Equivalent volume of deionized water, 0.1 mL/mouse, p.o.), standard drug (Diclofenac sodium, 10 mg/kg, i.p.) or MEDS (100, 200 and 400 mg/kg, p.o.) and then the writhing was induced by the injection of 0.6% acetic acid 15 min after drug and 30 min after oral administration of MEDS, respectively. 5 min after the injection of acetic acid, the mice were observed and the number of writhing was counted for 30 min [38]. The contractions of the stomach, elongation of the body, twisting of the trunk and pelvis finishing with the extension of the limbs were considered as complete writhing. Antinociceptive activity was calculated as the percentage inhibition of abdominal constriction.

#### Formalin test

The method was used as narrating by Santos and Calixto and Santos et al. [39, 40] with minor modification. The animals were arranged into five groups (*n* = 5). The control group received deionized water orally at the volume of 0.1 mL/mouse 30 min before the experiments. Twenty microliters of 2.5% formalin (in deionized water, subplantar) was injected subcutaneously into the right hind paw 1 h after MEDS treatment (100, 200 and 400 mg/kg, p.o.) and 15 min after injection of diclofenac sodium (10 mg/kg, i.p.) of the mice. The time spent licking and biting the injected paw was measured as an indicator of pain response. Responses were measured for 5 min subsequent to formalin injection (First phase, neurogenic) and 15–30 min after formalin injection (Second phase, inflammatory). Antinociceptive activity was calculated as the percentage inhibition of licking time.

### Glutamate-induced nociception

The method was used similarly to the previously described by Beirith et al. [41]. Mice were divided into five groups each containing five mice. A volume of 20 μL of glutamate solution (10 μmol per paw) was injected under the plantar surface of the right hind paw of the mice 30 min after MEDS (100, 200 and 400 mg/kg, p.o.) treatment, respectively and 15 min after injection of diclofenac sodium (10 mg/kg, i.p.). The control group received deionized water orally at the volume of 0.1 mL/mouse 30 min before the experiments. The mice were observed separately for 15 min following glutamate injection. The number of licking of its injected paw was indicative of nociception.

### Cinnamaldehyde-induced nociception

In order to provide more direct evidence, concerning the participation of TRPA1 in the effect of the MEDS, investigated its antinociceptive effect in cinnamaldehyde- induced licking test in the mouse paw [42]. The experimental animals were randomly divided into five groups consisting of 5 mice in each. After an adjustment period (20 min), 20 μl of cinnamaldehyde (10 nmol/paw) was injected into the ventral surface of the right hind paw of the mice 60 min after MEDS (100, 200 and 400 mg/kg, p.o.) treatment and 15 min after injection of diclofenac sodium (10 mg/kg, i.p.). The control group received deionized water orally at the volume of 0.1 mL/mouse 30 min before the experiments. Mice were observed from 0 to 5 min. (Neurogenic phase) and considered as indicative of nociception.

### Analysis of the possible mechanism of action of MEDS

#### Involvement of opioid system

The possible connection of the opioid system in the antinociceptive effect of MEDS was examined by injecting naloxone hydrochloride (2 mg/kg, i.p.), a non-selective opioid receptor antagonist, and 15 min prior to the administration of either morphine or MEDS (100, 200 and 400 mg/kg, p.o.). Then, the hot plate and tail immersion latencies were measured at 0, 30, 60, 90, and 120 min with the same cut off time of the 20s for the protection of animals [43].

#### Involvement of cyclic guanosine monophosphate (cGMP) pathway

To investigate the possible involvement of cGMP pathway to the antinociceptive effect of MEDS, mice were pre-treated with methylene blue (20 mg/kg), a non-specific inhibitor of nitric oxide/guanylyl cyclase, intraperitonially 15 min before the administration of diclofenac sodium or MEDS (100, 200 and 400 mg/kg, p.o.). Then, the nociceptive responses against 0.6% acetic acid injection were seen for 30 min, opening from 5 min post injection. The numbers of abdominal writhing were counted as the symptom of pain behavior [44, 45].

#### Involvement of ATP-sensitive K^+^ channels pathway

The possible contribution of K^+^ channels in the antinociceptive effect of MEDS was evaluated by using the method described by Mohamad et al. with slide modification [46]. The mice were pre-treated with glibenclamide (10 mg/kg), an ATP-sensitive K^+^ channel inhibitor, intraperitoneally 15 min before the administration of either diclofenac sodium or MEDS (100, 200 and 400 mg/kg, p.o.). The mice were challenged with i.p. injection of 0.6% acetic acid, 30 min post-treatment. Subsequent the injection of acetic acid, the mice were immediately placed in a circle box and the number of writhing was recorded for 30 min, starting from 5 min post injection.

### Statistical analysis

The results are presented as mean ± SEM. The statistical analysis was performed using one-way analysis of variance (ANOVA) followed by Dunnett’s post hoc test as appropriate using SPSS 18.00 software. Differences between groups were considered significant at a level of *p* < 0.001.

## Results

### Phytochemical screening

The phytochemical screening of the crude extract of *D. sissoo* revealed the presence of flavonoids, tannins, cardiac glycosides, carbohydrates, proteins, and terpenoids (Table 1).

**Table 1:** Preliminary qualitative phytochemical screening of methanolic extract of *D. sissoo* (MEDS)

Plant constituents	Inference
Alkaloids	-
Flavonoids	+++
Saponins	-
Tannins	++
Cardiac Glycosides	+
Carbohydrates	+
Reducing Sugars	-
Proteins	+
Glucosides	-
Terpenoids	+
Steroids	-

+++: Most prominent; ++: Moderate; +: Lower; -: Absence

### Acute toxicity test

In the acute toxicity study, all mice survived and did not manifest any sign of toxicity and abnormality at the doses of 50–3000 mg/kg. After oral administration, there was no behavioral or body weight changes and no abnormal signs were observed for a period of 7 days. Therefore, it can be indicated that MEDS has low toxicity profile and the LD^50^ is more than 3000 mg/kg.

### Hot plate test

**Fig. 1:**
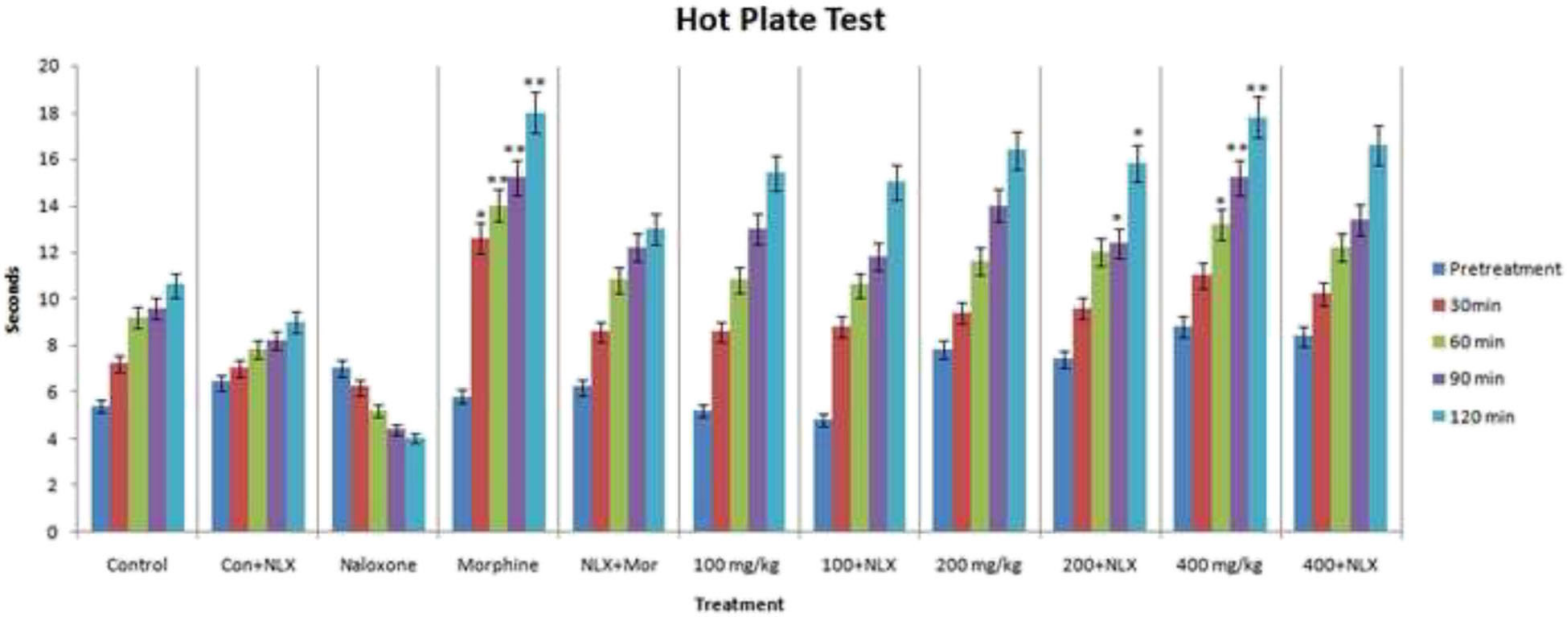
Antinociceptive effect of *D. sissoo* leave extract, morphine and reversal effect of naloxone in hot plate test. Values are presented as mean ± SEM (*n* = 5). ** *p* < 0.01 compared with the control group (ANOVA followed by post hoc Dunnett’s test)

As presented in Fig. 1 and Table 2, MEDS showed a significant antinociceptive effect at the doses of 200 mg/kg and 400 mg/kg (*p* < 0.05). The administration of naloxone did not cause any significant attenuation of antinociceptive activity of MEDS. Moreover, administration of morphine at the dose of 5 mg/kg confirmed a significant antinociceptive effect (*p* < 0.01) when compared with control group (Deionized water).

**Table 2:** Antinociceptive effect of *D. sissoo* leave extract, morphine and reversal effect of naloxone in hot plate test

Treatment		Dose (mg/kg)	Latency period (s) (% MPE) Pretreatment	30 min	60 min	90 min	120 min
Treatment without Naloxone	Control	0.1 mL/mouse	5.40 ± 1.20	7.20 ± 1.56	9.20 ± 0.86	9.60 ± 1.07	10.60 ± 1.07
	Morphine	5	5.82 ± 0.86	12.60 ± 0.92* (42.19)	14.00 ± 0.70** (44.44)	15.20 ± 0.86** (53.84)	18.00 ± 1.14** (78.72)
	MEDS	100	5.20 ± 0.97	8.60 ± 2.01 (10.93)	10.80 ± 0.86 (14.81)	13.00 ± 1.00 (32.69)	15.40 ± 1.28 (51.06)
	MEDS	200	7.80 ± 1.46	9.40 ± 0.92 (17.18)	11.60 ± 1.20 (22.22)	14.00 ± 1.22* (42.30)	16.40 ± 1.53* (61.70)
	MEDS	400	8.80 ± 1.24	11.00 ± 0.70 (29.68)	13.20 ± 1.06* (37.03)	15.20 ± 1.15** (53.84)	17.80 ± 1.53** (76.59)
Treatment with Naloxone	NLX	2	7.00 ± 0.70	6.20 ± 0.58	5.20 ± 0.66	4.40 ± 0.51	4.00 ± 0.70
	NLX+ Control	2 + 0.1 mL/mouse	6.64 ± 0.74	7.00 ± 0.70	7.80 ± 0.86	8.20 ± 0.86	9.00 ± 0.89
	NLX+ Morphine	2 + 5	6.20 ± 0.58	8.60 ± 0.51 (10.93)	10.80 ± 0.73 (14.81)	12.20 ± 0.86 (25)	13.00 ± 1.14 (25.53)
	NLX+ MEDS	2 + 100	4.80 ± 0.58	8.80 ± 0.66 (12.50)	10.60 ± 0.51 (12.96)	11.80 ± 0.80 (21.15)	15.00 ± 0.70 (46.80)
	NLX+ MEDS	2 + 200	7.40 ± 1.20	9.60 ± 0.67 (18.75)	12.00 ± 0.70 (25.92)	12.40 ± 0.92 (26.92)	15.80 ± 1.59 (55.31)
	NLX+ MEDS	2 + 400	8.40 ± 0.81	10.20 ± 0.58 (23.43)	12.20 ± 0.37 (27.77)	13.40 ± 0.92 (36.52)	16.60 ± 1.43 (63.82)

Values are presented as mean ± SEM (*n* = 5). *MEDS* Methanolic extract of *D. sissoo*, *NLX* Naloxone

**p < 0.01 compared with the control group (Dunnett’s test)

*p < 0.05 compared with the control group (Dunnett’s test)

### Tail immersion test

**Fig. 2:**
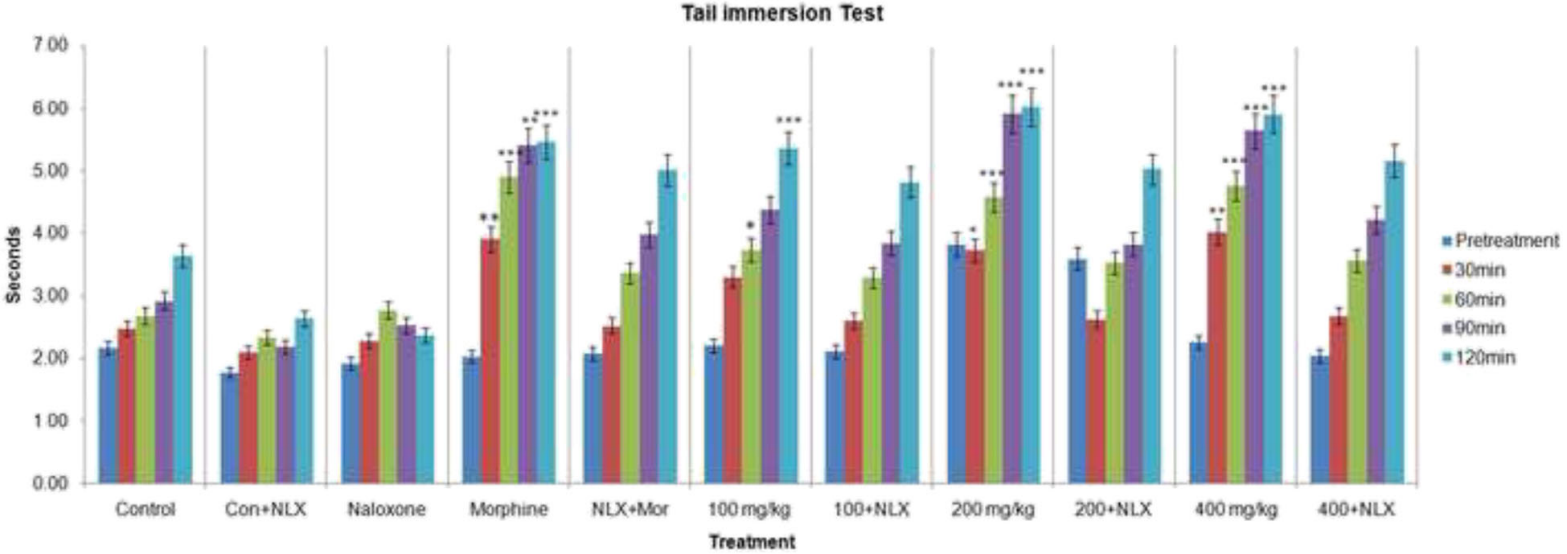
Antinociceptive effect of *D. sissoo* leave extract, morphine and reversal effect of naloxone in tail immersion test. Values are presented as mean ± SEM (*n* = 5). *** *p* < 0.001 compared with the control group (ANOVA followed by post hoc Dunnett’s test)

The tail-immersion test results asserted significant antinociceptive effect (*p* < 0.001) compared with control, at the doses of 200 and 400 mg/kg. The antinociceptive effect of 100, 200, and 400 mg/ kg of MEDS were comparable to that of the reference drug (Fig. 2 and Table 3). The antinociceptive activity of morphine used in this test was antagonized by naloxone. The effect of naloxone was not remarkable at any of the doses. A significant antinociceptive effect was produced by morphine (*p* < 0.001) when compared with control group (Deionized water).

**Table 3:** Antinociceptive effect of *D. sissoo* leave extract, morphine and reversal effect of naloxone in tail immersion test

Treatment		Dose (mg/kg)	Latency period (s) (% MPE) Pretreatment	30 min	60 min	90 min	120 min
Treatment without Naloxone	Control	0.1 mL/mouse	2.16 ± 0.23	2.48 ± 0.16	2.68 ± 0.19	2.92 ± 0.12	3.64 ± 0.14
	Morphine	5	2.03 ± 0.15	3.90 ± 0.18** (8.10)	4.90 ± 0.18*** (12.81)	5.40 ± 0.40** (14.51)	5.46 ± 0.25***(11.12)
	MEDS	100	2.20 ± 0.20	3.30 ± 0.12 (4.68)	3.73 ± 0.15* (6.08)	4.37 ± 0.21 (8.50)	5.36 ± 0.24***(10.51)
	MEDS	200	3.81 ± 0.18	3.72 ± 0.27* (7.10)	4.58 ± 0.45*** (10.94)	5.91 ± 0.68*** (17.49)	6.02 ± 0.14***(14.53)
	MEDS	400	2.26 ± 0.09	4.02 ± 0.43** (8.80)	4.76 ± 0.23*** (12.00)	5.64 ± 0.37*** (15.92)	5.90 ± 0.18***(13.81)
Treatment with Naloxone	NLX	2	1.92 ± 0.13	2.28 ± 0.16	2.77 ± 0.15	2.53 ± 0.16	2.37 ± 0.18
	NLX+ Control	2 + 0.1 mL/mouse	1.77 ± 0.10	2.09 ± 0.16	2.33 ± 0.14	2.18 ± 0.11	2.63 ± 0.11
	NLX+ Morphine	2 + 5	2.08 ± 0.05	2.52 ± 0.13 (0.22)	3.36 ± 0.18 (3.92)	3.98 ± 0.37 (6.20)	5.01 ± 0.30 (8.36)
	NLX+ MEDS	2 + 100	2.11 ± 0.14	2.60 ± 0.11 (0.68)	3.29 ± 0.15 (3.52)	3.84 ± 0.39 (5.38)	4.82 ± 0.09 (7.21)
	NLX+ MEDS	2 + 200	3.59 ± 1.14	2.63 ± 0.18 (0.85)	3.52 ± 0.29 (4.84)	3.82 ± 0.37 (5.24)	5.02 ± 0.13 (8.43)
	NLX+ MEDS	2 + 400	2.04 ± 0.07	2.68 ± 0.27 (1.14)	3.56 ± 0.34 (5.09)	4.21 ± 0.25 (7.56)	5.16 ± 0.16 (9.29)

Values are presented as mean ± SEM (*n* = 5). *MEDS* Methanolic extract of *D. sissoo*, *NLX* Naloxone

*** *p* < 0.001 compared with the control group (Dunnett’s test)

** *p* < 0.01 compared with the control group (Dunnett’s test)

* *p* < 0.05 compared with the control group (Dunnett’s test)

### Acetic acid-induced writhing test

**Fig. 3:**
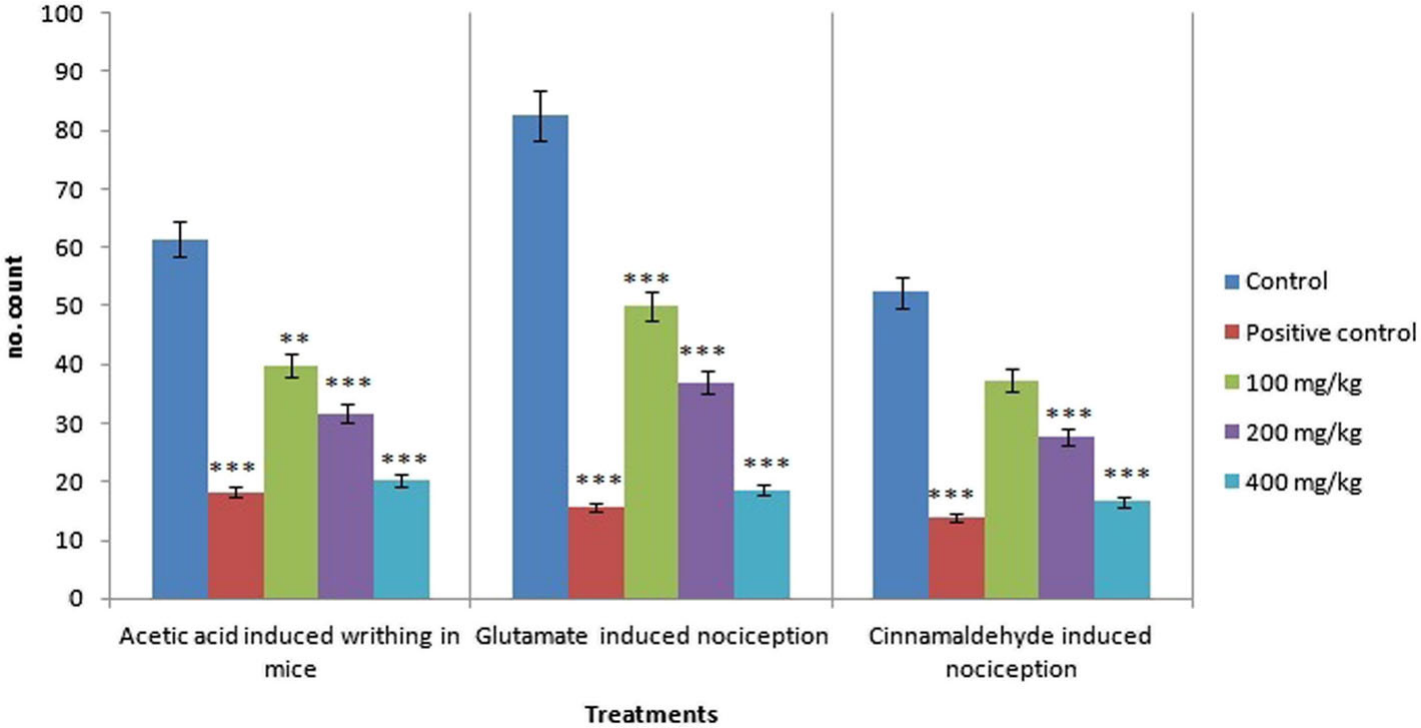
Antinociceptive effects of *D. sissoo* leave extract in acetic acid-induced writhing, glutamate-induced nociception and cinnamaldehyde-induced nociception tests. All values are presented as mean ± SEM (*n* = 5). *** *p* < 0.001 compared with the control group (ANOVA followed by post hoc Dunnett’s test)

The effect of administration of MEDS using the abdominal constriction test in mice is shown in Fig. 3 and Table 4. It was found that MEDS was able to inhibit significantly the nociceptive effects induced by acetic acid compared to the control group (Deionized water) at the doses of 100, 200, and 400 mg/kg, respectively (*p* < 0.001). The percentage inhibition of constrictions was calculated as 70.36% (Diclofenac sodium, 10 mg/kg), 35.18% (MEDS, 100 mg/kg), 48.53% (MEDS, 200 mg/kg), and 67.10% (MEDS, 400 mg/kg) (Table 4).

**Table 4:** Antinociceptive effects of *D. sissoo* leave extract in acetic acid-induced writhing test

Treatment	Dose (mg/kg)	Number of writhing	Inhibition (%)
Control	0.1 mL/ mouse	61.4 ± 1.29	
Diclofenac sodium	10	18.2 ± 2.25***	70.36
MEDS	100	39.8 ± 1.78**	35.18
MEDS	200	31.6 ± 1.69***	48.53
MEDS	400	20.2 ± 1.60***	67.10

Values are presented as mean ± SEM (*n* = 5). *MEDS* Methanolic extract of *D. sissoo*

*** *p* < 0.001 compared with the control group (Dunnett’s test)

** *p* < 0.01 compared with the control group (Dunnett’s test)

### Formalin test

**Fig. 4:**
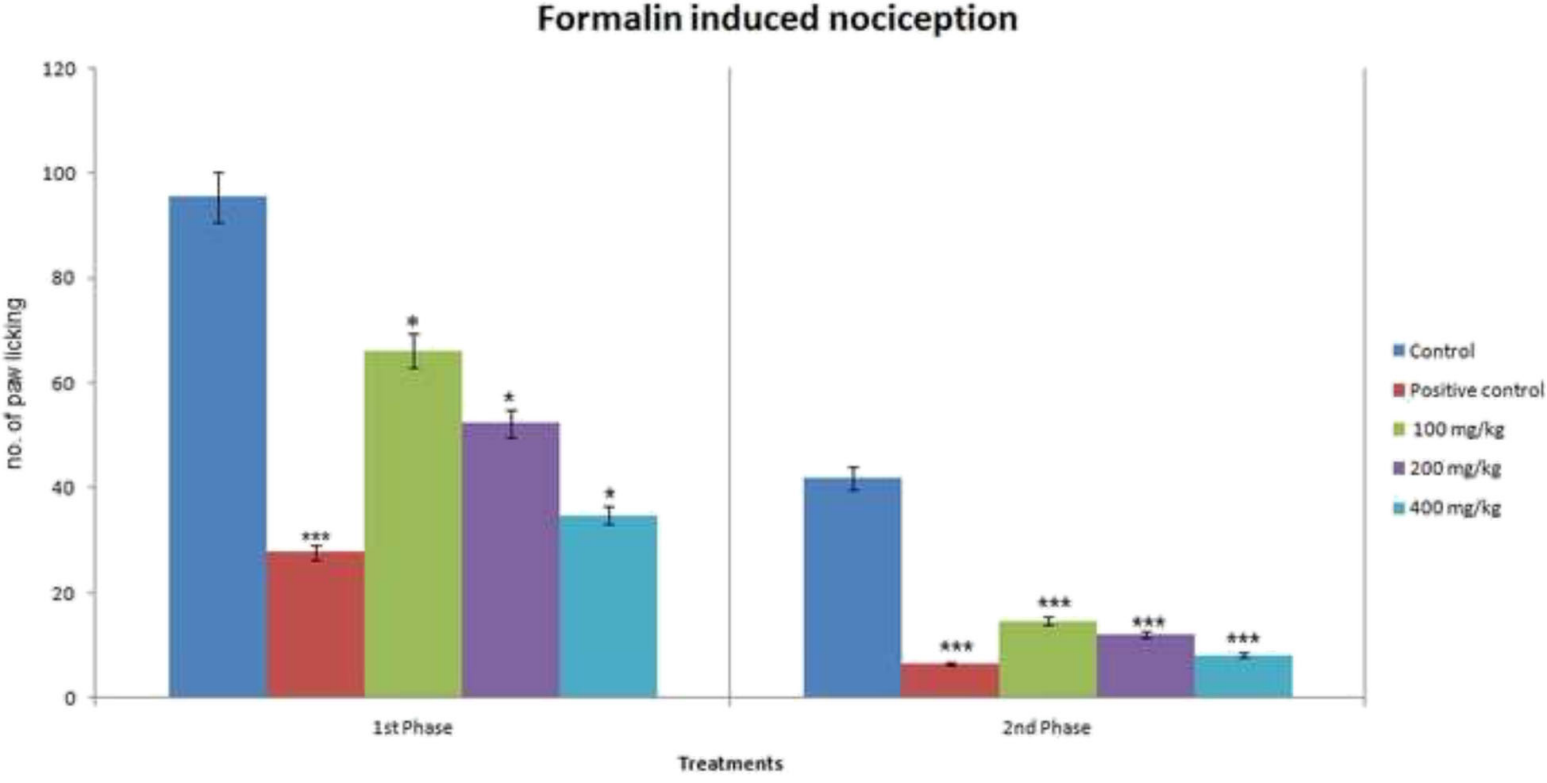
Antinociceptive effects of *D. sissoo* leave extract in formalin-induced nociception. Values are presented as mean ± SEM (*n* = 5). *** *p* < 0.001 compared with the control group (ANOVA followed by post hoc Dunnett’s test)

MEDS produced a dose-related inhibition of formalin-induced nociception and caused significant inhibition of both neurogenic (0–5 min) and inflammatory (15–30 min) phases of formalin-induced licking test at the doses of 100, 200, and 400 mg/kg when compared with control group (Deionized water) (Fig. 4 and Table 5). However, its antinociceptive effect was more pronounced in the second phase of this model of pain. Diclofenac sodium (10 mg/kg, i.p.) significantly reduced formalin-induced nociception in both phases (*p* < 0.001).

**Table 5:** Antinociceptive effects of *D. sissoo* leave extract in formalin-induced nociception

Treatment	Dose (mg/kg)	Licking of the hind paw			
		Early phase (0–5 min)	Inhibition (%)	Late phase (15–30 min)	Inhibition (%)
Control	0.1 mL/ mouse	95.40 ± 1.33	–	41.80 ± 2.15	–
Diclofenac sodium	10	27.80 ± 2.73***	70.86	6.60 ± 0.51***	84.21
MEDS	100	66.20 ± 1.98*	30.60	14.60 ± 1.75***	65.05
MEDS	200	52.40 ± 0.87*	45.07	12.00 ± 1.00***	71.29
MEDS	400	34.80 ± 1.20*	63.52	08.20 ± 0.73***	80.38

Values are presented as mean ± SEM (*n* = 5). *MEDS* Methanolic extract of *D. sissoo*

*** *p* < 0.001 compared with the control group (Dunnett’s test)

* *p* < 0.05 compared with the control group (Dunnett’s test)

### Glutamate-induced nociception

The antinociceptive activity induced by oral administration of MEDS was dose-dependent. It showed that MEDS at the doses of 100, 200, and 400 mg/kg produced significant prohibition of the glutamate-induced nociception test (Fig. 3 and Table 6). Diclofenac sodium (10 mg/kg) was used as a standard drug, which showed 81.11% inhibition of licking as compared to the control group. All treatments displayed significant antinociceptive activity (*p* < 0.001) compared together with the control group (Deionized water).

**Table 6:** Antinociceptive effects of *D. sissoo* leave extract in glutamate-induced nociception

Treatment	Dose (mg/kg)	Licking time (s)	Inhibition (%)
Control	0.1 mL/ mouse	82.60 ± 1.72	–
Diclofenac sodium	10	15.60 ± 1.08***	81.11
MEDS	100	50.00 ± 1.61***	39.46
MEDS	200	37.00 ± 1.30***	55.20
MEDS	400	18.60 ± 1.36***	77.48

Values are presented as mean ± SEM (*n* = 5). *MEDS* Methanolic extract of *D. sissoo*

*** *p* < 0.001 compared with the control group (Dunnett’s test)

### Cinnamaldehyde-induced nociception

The result of cinnamaldehyde-induced nociception showed that administration of MEDS at 100, 200, and 400 mg/kg dose produced dose-dependent inhibition of the cinnamaldehyde-induced neurogenic nociception with the percentage of inhibition of 28.62%, 47.32% and 68.32%, respectively (Fig. 3 and Table 7). Only 100 mg/kg MEDS treatment showed no significant difference compared with the control group (Deionized water), at the same time as the rest showed significant antinociceptive activity (*p* < 0.001).

**Table 7:** Antinociceptive effects of *D. sissoo* leave extract in cinnamaldehyde-induced nociception

Treatment	Dose (mg/kg)	Licking time (s)	Inhibition (%)
Control	0.1 mL/ mouse	52.40 ± 1.54	
Diclofenac sodium	10	13.80 ± 1.53***	73.66
MEDS	100	37.40 ± 0.93	28.62
MEDS	200	27.60 ± 1.66***	47.32
MEDS	400	16.60 ± 1.86***	68.32

Values are presented as mean ± SEM (*n* = 5). *MEDS* Methanolic extract of *D. sissoo*

*** *p* < 0.001 compared with the control group (Dunnett’s test)

### Involvement of cyclic guanosine monophosphate (cGMP) pathway

**Fig. 5:**
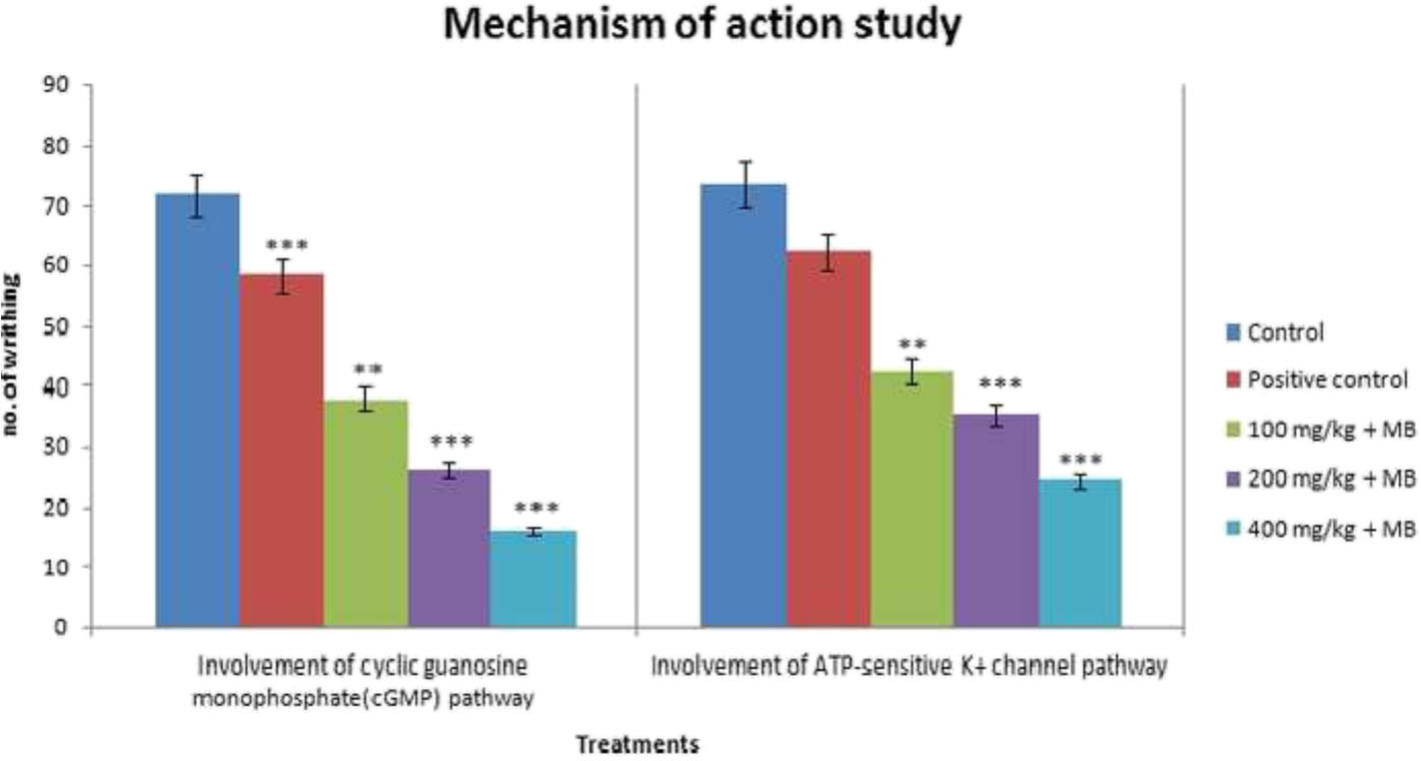
Effects of *D. sissoo* leave extract on involvement of cyclic guanosine monophosphate (cGMP) pathway and ATP-sensitive K^+^ channel pathway. Values are presented as mean ± SEM (*n* = 5). *** *p* < 0.001 compared with the control group (ANOVA followed by post hoc Dunnett’s test)

The present results showed at the effects of 100, 200, and 400 mg/ kg MEDS and methylene blue (20 mg/kg) treatments. Methylene blue administration alone significantly inhibited acetic acid-induced abdominal writhing (Fig. 5 and Table 8). Given together, methylene blue significantly (*p* < 0.001) increased MEDS (200, and 400 mg/kg) induced antinociception compared to the control group (Deionized water).

**Table 8:** Effects of *D. sissoo* leave extract on involvement of cyclic guanosine monophosphate (cGMP) pathway

Treatment	Dose (mg/kg)	Number of writhing	Inhibition (%)
Control	0.1 mL/ mouse	71.74 ± 1.77	–
Methylene Blue (MB)	20	58.20 ± 2.89***	18.87
MEDS + MB	100 + 20	37.90 ± 0.96**	47.17
MEDS + MB	200 + 20	26.10 ± 1.69***	63.62
MEDS + MB	400 + 20	15.90 ± 1.52***	77.84

Values are presented as mean ± SEM (*n* = 5). *MEDS* Methanolic extract of *D. sissoo*

*** *p* < 0.001 compared with the control group (Dunnett’s test)

** *p* < 0.01 compared with the control group (Dunnett’s test)

### Involvement of ATP-sensitive K^+^ channels pathway

The present study seemed at the effects of 100, 200, and 400 mg/ kg MEDS and glibenclamide (10 mg/kg) treatments. It was found out that glibenclamide (10 mg/kg) administration alone did not alter abdominal writhing count when assessed through the injection of 0.6% acetic acid (Fig. 5 and Table 9). When given together, the antinociceptive activity of MEDS was noticeably decreased by glibenclamide at the doses of 200, and 400 mg/kg, respectively.

**Table 9:** Effects of *D. sissoo* leave extract on involvement of ATP-sensitive K^+^ channel pathway

Treatment	Dose (mg/kg)	Number of writhing	Inhibition (%)
Control	0.1 mL/ mouse	73.4 ± 1.71	–
Glibenclamide (GB)	10	62.3 ± 1.11	15.12
MEDS + GB	100 + 10	42.3 ± 2.07**	42.37
MEDS + GB	200 + 10	35.3 ± 1.45***	51.91
MEDS + GB	400 + 10	24.2 ± 2.02***	67.03

Values are presented as mean ± SEM (*n* = 5). *MEDS* Methanolic extract of *D. sissoo*

*** *p* < 0.001 compared with the control group (Dunnett’s test)

** *p* < 0.01 compared with the control group (Dunnett’s test)

## Discussion

The study investigated the antinociceptive activity of MEDS in classical pharmacological models of pain. The findings of this study specify the peripheral and central antinociceptive effect of MEDS at different doses in mice models. The hot plate latency time was dose-dependently enhanced by MEDS (*p* < 0.05) at different doses suggest that the central antinociceptive activity of MEDS (Fig. 1 and Table 2). The effect is further supported by the results viewed in the tail immersion test (Fig. 2 and Table 3), as the tail removal response in hot water-induced pain is selective just for centrally acting analgesics, while the peripherally acting agents are inactive [47]. The results obtained from these two models suggest that antinociceptive effect of MEDS was reversed by naloxone, a non-selective opioid receptor antagonist, against the antinociceptive effect of MEDS. The central antinociceptive effect of MEDS may arise through opioid receptors of spinal as well as a supraspinal system. Both tail immersion and hot plate tests are based on measuring the response of the animal to thermal stimuli where the tail immersion check a spinal reflex, and the hot plate is used for supraspinal reflex [48]. Reach an agreement with this suggestion that μ2- and δ-opioid receptors are involved in spinal mechanism, while μ1/μ2-opioid receptors may mediate principally supraspinal analgesia [49]. This clearly suggests that the involvement of the activation of opioid receptors in the antinociceptive action of MEDS.

In the acetic acid-induced writhing test, a dose-dependent antinociceptive effect was observed after oral administration of MEDS (Fig. 3 and Table 4). This test has been used as a screening tool for assessing of the central and peripheral antinociceptive activity [50]. In general, acetic acid causes pain by liberating endogenous substances such as serotonin histamine, prostaglandins (PGs), bradykinins and substance P. Local peritoneal receptors are postulated to be occupied in the abdominal constrictions response [51]. Intraperitoneal administration of acetic acid causes an increment in cyclooxygenase (COX), lipoxygenase (LOX), prostaglandins (PGs), histamine, serotonin, bradykinin, substance P, IL-1β, IL-8,TNF-α in the peripheral tissue fluid. These mediators cause the stimulation of primary afferent nociceptors entering dorsal horn of the central nervous system [52]. The release of these inflammatory mediators is thought to contribute to increased blood-brain barrier (BBB) permeabilization or interruption [53]. Moreover, injection of pain inducing agent like acetic acid is also responded to enhance vasodilation and vascular fluid permeability and these events were reversed by plant extracts [54]. From a mechanistic point of view, the lack of specificity in acetic acid-induced writhing test suggesting the participation of different nociceptive mechanisms in the reduction of muscular constriction such as sympathetic system throughout the release of biogenic amines, cyclooxygenases and their metabolites inhibition and through opioid receptors mechanisms [55]. Therefore, the inhibition of writhing response in the present study clearly indicated the peripheral antinociceptive effect of MEDS in addition to its central effect.

Formalin-induced test is a pain-related licking response of the injected paw in two distinct phases. The first phase is presented by neurogenic pain caused by direct chemical stimulation of nociceptors. The second phase is marked by inflammatory pain triggered by a fusion of stimuli-inflammation of the peripheral tissues and mechanisms of central sensitization. In this latter part, different mediators are involved, as excitatory amino acids, neuropeptides, nitric oxide, PGE_2_, and kinins [56]. Our present results showed that the number of paw licking was significantly reduced by MEDS in both neurogenic and inflammatory pain responses (*p* < 0.001) in a dose dependant manner (Fig. 4 and Table 5). However, the effect was more assert in the late phase. Centrally acting analgesic drugs inhibit both the phases of formalin test, while peripherally acting analgesics restrict only the late phase responses. Diclofenac sodium significantly suppressed the late phase pain response. The first phase finding of formalin test further confirms the central antinociceptive effect of MEDS that we have observed in the hot plate and tail immersion tests. The late phase response as the antinociceptive effect observed in acetic acid-induced writhing test is due to this inhibition of the inflammatory mediators [57].

Phytochemical screening exposed that flavonoids, tannins, cardiac glycosides, carbohydrates, proteins, and terpenoids are present in D. *sissoo*. The MEDS showed significant and dose-dependent antinociceptive activity due to the presence of flavonoids [23]. Flavonoids have been found to suppress the intracellular Ca^2+^ ion elevation in a dose-dependent manner, as well as possible the release of proinflammatory mediators such as TNF-α [58]. Flavonoids may enhance the amount of endogenous serotonin or may act together with 5-HT_2_ and 5-HT_3_ receptors, which may be involved in the mechanism of central pain reliever activity [59]. There are also reports on the role of flavonoid in analgesic activity principally by targeting prostaglandins [60]. Moreover, MEDS showed significant analgesic activity in the whole experimental model, which may be due to its high flavonoid. Furthermore, flavonoids have the capability to inhibit ecosanoid biosynthesis. Ecosanoids, such as prostaglandins are involved in different immunological responses and are the products of the cyclooxygenase and lipoxygenase pathways [61]. Tannins are also invented to have a contribution in antinociceptive activity [62]. Therefore, it can be agreed that cyclooxygenase (COX) inhibitory activity alongside with antioxidant activity may reduce the production of arachidonic acid from phospholipids or may reduce the enzyme system responsible for the synthesis of prostaglandins and finally relieve pain-sensation.

In another experiment to determine the role of the glutamatergic system in the modulation of MEDS antinociception, the extract was treated with the glutamate-induced paw-licking test. Previous reports have shown that the glutamate and glutamatergic receptors are very important in the peripheral, spinal, and supraspinal nociceptive neurotransmission [63–65], which is greatly mediated by both N-methyl-D-aspartate (NMDA) and non-NMDA receptors, as well as possible by the release of nitric oxide and nitric oxide-related substances [41]. On the other hand, N-methyl-D-aspartate receptor antagonists have been proven to inhibit the spread of pain sensation and to decrease the hyperactive excitability of spinal cord neurons triggered by C-fiber stimulation [66, 67]. In addition, activation of glutamate receptors also has been shown to contribute to the continuation of peripheral nociceptive processes that are connected with inflammatory pain [68], which is concurrent with the report that application of glutamate receptor antagonist obstructed the inflammatory phases of the formalin test. Based on our findings, glutamatergic system involved in the modulation of MEDS antinociception (Fig. 3 and Table 6).

In addition, recent studies have been assessed the cinnamaldehyde-induced nociception, to investigate the participation of TRPA1 receptor in the antinociceptive effect of MEDS. It has shown that cinnamaldehyde activates primary afferent sensory neurons through a direct action on TRPA1, a member of the Transient Receptor Potential family (TRP) of cation channels that are highly expressed by a subset of C-fibre nociceptors [69]. Currently, it was demonstrated that intraperitoneal administration of cinnamaldehyde, a TRPA1 agonist receptor, to mice produced a dose-dependent regular nociception [70]. Our results show that MEDS significantly reduced the cinnamaldehyde-induced pain (Fig. 3 and Table 7). This result indicates that MEDS probably interacts with the TRPA1 receptor located in C-fibres reducing the cinnamaldehyde -induced nociception.

The present study also investigated the possible participation of cGMP pathway on the antinociceptive activity of MEDS (Fig. 5 and Table 8). Physiological functions as pain and analgesia are influenced by the cellular level of cGMP-regulated by the action of sGC interceded by nitric oxide (NO) [71]. The cGMP pathway depends on the synthesis and release of nitric oxide triggered by the activation of nitric oxide-synthase (NOS), which then activates the guanylyl synthase resulted in the formation of cGMP, the most monumental messenger of the system [72]. Therefore, cGMP seems to be very significant for the functioning of up or down regulation of nociceptor. Intracellular cGMP concentrations are regulated by the action of GCs and by the rate of degradation by cGMP-specific phosphodiesterases. It has been reported that the action of cGMP on the ion channels may be direct or through the activation of protein kinases and phosphodiesterases [73]. To detect the feasible involvement of cGMP in MEDS induced antinociception, methylene blue (MB), aguanylyl cyclase and/or nitric oxide synthase inhibitor, was administered prior to inducing nociception with the intraperitoneal injection of acetic acid. The result presents that the pre-treatment with methylene blue significantly reduced the nociception caused by acetic acid and enhanced the antinociceptive effect exerted by MEDS. It has been suggested that MB promotes antinociceptive activity by inhibiting peripheral NOS and sGC, resulting in NO interference [70]. As pre-treatment with MB and subsequent administration of MEDS at all doses increased antinociceptive effect in acetic acid-induced writhing test in mice. These results suggest that the cGMP pathways have been involved in the antinociceptive effects of the MEDS in the acetic acid-induced nociception in mice model.

The results also point out that the antinociceptive effect of MEDS might involve the participation of the ATP–sensitive K^+^ channel pathway; glibenclamide, an ATP–sensitive K^+^ channel antagonist, could partially reverse the antinociceptive activity of MEDS (Fig. 5 and Table 9). Previous studies report specific blockade of ATP-sensitive K^+^ channel by glibenclamide while not affecting other types of K^+^ channel like Ca^2+^ activated and voltage-gated K^+^ channels [74, 75]. The antinociceptive action of MEDS might relate to ATP–sensitive K^+^ channel opening and the subsequent efflux of K^+^ ions and membrane repolarization or hyperpolarization [76].

## Conclusions

It can be concluded that MEDS possesses significant antinociceptive activity in both chemical and heat-induced pain models in mice. The antinociceptive effect of MEDS is most likely mediated via inhibition of peripheral mediators and central inhibitory mechanisms. These results support the traditional use of this plant in different painful conditions. Further investigations are required to perceive the mechanisms of action of MEDS and to identify the active constituents that may be used as a lead compound for new drug development.

## Abbreviations

BBB: Blood brain barrier
cGMP: Cyclic guanosine monophosphate
ICDDR, B: International Center for Diarrheal Disease and Research, Bangladesh
MEDS: Methanolic extract of *D. sissoo*
TRP: Transient Receptor Potential
